# Identification of Gut Microbiota and Metabolites Signature in Patients With Irritable Bowel Syndrome

**DOI:** 10.3389/fcimb.2019.00346

**Published:** 2019-10-18

**Authors:** Shengtao Zhu, Si Liu, Hengcun Li, Zheng Zhang, Qian Zhang, Lei Chen, Yu Zhao, Yang Chen, Junchao Gu, Li Min, Shutian Zhang

**Affiliations:** ^1^Department of Gastroenterology, Beijing Friendship Hospital, Capital Medical University, National Clinical Research Center for Digestive Disease, Beijing Digestive Disease Center, Beijing Key Laboratory for Precancerous Lesion of Digestive Disease, Beijing, China; ^2^MOE Key Laboratory of Bioinformatics, Bioinformatics Division and Center for Synthetic and Systems Biology, Department of Automation, BNRist, Tsinghua University, Beijing, China; ^3^Beijing Tropical Medicine Research Institute, Beijing Friendship Hospital, Capital Medical University, Beijing, China

**Keywords:** irritable bowel syndrome (IBS), gut microbiota, gut metabolites, severity of IBS, 16S rDNA amplicon sequencing, correlation analysis

## Abstract

**Background and Aims:** Irritable bowel syndrome (IBS) is a common functional gastrointestinal disorder. However, the underlying mechanism of IBS is not fully understood. The aim of this study was to investigate potential mechanism and novel biomarkers of IBS through evaluation of the metabolomic and microbiologic profile.

**Methods:** Fecal samples were collected from 15 irritable bowel syndrome patients and 15 healthy controls. By using gas chromatography coupled to time-of-flight mass spectrometry (GC-TOFMS) and 16S rDNA amplicon sequencing, fecal metabolites and microbiota of healthy controls and the IBS patients were measured.

**Results:** IBS patients had a significantly differential metabolite profile as compared to healthy controls, and 4 clusters with 31 metabolites, including a group of amino acids and fatty acids, were significantly up-regulated as compared to the healthy controls. In addition, 19 microbes were significantly up-regulated, and 12 microbes were down-regulated in the IBS group, when compared with the healthy controls. Some clusters of fecal metabolites or microorganisms were significantly correlated with the severity of IBS symptoms, such as the frequency of abdominal pain/discomfort and the number of bowel movements. Correlation of the metabolite levels with abundances of microbial genera showed some statistically significant metabolite-microbe associations. Four differentially abundant amino acids clustered together were positively correlated with some microbes, including *Lachnospira, Clostridium*, and so on.

**Conclusion:** The finding of this study puts a global perspective on metabolomics and microbiota profiling in IBS patients and provides a theoretical basis for future research on pathophysiology of IBS.

## Introduction

Irritable bowel syndrome (IBS) is one of the most common functional bowel disorders, affecting 10–15% of the general population worldwide (Lovell and Ford, 2012a,b). It is characterized by chronic and recurrent abdominal pain/discomfort and disordered bowel habits in the absence of other organic gastrointestinal disease (Mayer, 2008; Ford et al., 2017). IBS can be clinically subtyped into IBS with constipation (IBS-C), IBS with diarrhea (IBS-D) and mixed IBS (IBS-M) (Guilera et al., 2005; Oswiecimska et al., 2017). In addition, IBS patients appeared to have a higher frequency of psychosocial stress, a lower quality of life and lower levels of work productivity (Drossman et al., 1988, 2000; Talley et al., 1997).

The detailed pathophysiology of IBS is unknown but is thought to be heterogeneous. Previous studies of IBS mainly focused on the altered gastrointestinal motility, increased gut sensitivity, brain-gut interaction, and psychosocial distress (Kellow and Phillips, 1987; Rogers et al., 1989; Mayer and Tillisch, 2011). In addition, dysregulated inflammation and immune function were also considered to play roles in the pathogenesis of IBS (Ohman and Simren, 2010).

Human gastrointestinal tract is a complex environment that includes a high diversity of inhabiting microorganisms, and complex interactions between microbes and the host (Yamashiro, 2017). Large number of studies have been focusing on the gut microenvironment in IBS patients. Many studies have reported different compositions of fecal microbiota between IBS patients and healthy controls (Si et al., 2004; Carroll et al., 2012). For example, Palva et al. have reported that IBS-D patients have decreased amounts of *Lactobacillus* spp. in the fecal samples and IBS-C patients have increased amounts of *Veillonella* spp. (Costello et al., 2009). In addition, the diverse community of microorganisms has significant impact on human metabolism (Belizario and Faintuch, 2018; O'Malley, 2019). Noorbakhsh et al. have reported that the metabolite concentrations of serum and urine in healthy group and IBS-D patients were significantly different at baseline (Noorbakhsh et al., 2018). Researches on complex metabolic interactions between microbes and host tissues have been proved to be critical to prevent various gut disorders, such as IBS and ulcerative colitis (UC) (Ghaisas et al., 2016; Lopetuso et al., 2018). Integrated bioinformatic analysis is emerging as an important method to investigate microbes and metabolites, which can provide us new insights into the relationship between metabolites and microbiota and reveal a novel therapeutic strategy of IBS.

In order to investigate the interactions between gut microbiota and metabolome in IBS, both metabolite or microbe profiling analysis and correlative microbe–metabolite analysis were used in this study to analyze fecal samples from IBS patients or healthy people.

## Materials and Methods

### Participants and Sample Collection

All participants were Chinese Han nationality. A total of 15 IBS patients meeting the Rome III criteria were recruited from the gastroenterology clinic in Beijing Friendship Hospital affiliated to Capital Medical University, Beijing, China. Fifteen healthy volunteers from physical examination center at Beijing Friendship Hospital were enrolled in the healthy control group These healthy volunteers had normal bowel movements without abdominal symptoms, coronary artery disease, inflammatory conditions and diabetes mellitus. Participants of IBS with diabetes, asthma, pregnancy, and earlier abdominal surgeries were excluded. Participants were asked not to take any antibiotics, eat spicy food, and smoke 4 weeks prior of sample collection. All participants have signed informed consent and the study was approved by the Ethics Committee of Beijing Friendship Hospital. Water ban was also required after midnight before collecting the samples in the morning. First early morning fecal samples were collected from each participant in sterile fecal specimen cups. Stool specimens were collected and handled by experienced clinicians and trained technicians. Each sample was divided into two tubes for metabolome and microbiota analysis and stored at −80°C until analysis.

### Microbial 16S rDNA Amplication Sequencing

By using a Power Soil® DNA Isolation Kit (MO BIO Laboratories, Carlsbad, CA), DNA was extracted from fecal pellets according to the manufacturer's instructions. The DNA samples were quantified by ultraviolet spectroscopy and stored at −80°C for further analysis. By using universal primers of U515(GTGCCAGCMGCCGCGGTAA)and E786 (GGACTACHVGGGTWTCTAAT), the V4 regions of the bacterial 16S rRNA gene were amplified. Individual amplification products were purified, barcoded and pooled to construct the sequencing library. Samples were sequenced with Illumina Miseq (Illumina HiSeq 2500) to generate pair-ended 150 × 150 reads. The sequenced raw data were then spliced and filtered to obtain the clean data. Thereafter, operational taxonomic units (OTUs) clustering and species classification were performed.

### Sample Preparation and Fecal Metabolite Profiling

Fecal samples from two different groups (IBS patients and healthy people) were stored at −80°C before the process. The experiment was performed by using test kit—MicrobioMET (Metabo-Profile, Shanghai, P.R. China). MicrobioMET is a commercial total solution including standardized, quality-controlled sample preparation, rapid GC-TOFMS analysis, data analysis software, and project report. Briefly, samples were thawed on ice-bath and weighed 50 mg for preparation. Materials were then homogenized with 1 M NaOH (300 μL) solution and centrifuged at 13,500 rpm at 4°C for 20 min. Each 200 μL supernatant was transferred into an autosampler vial. The residue was further exacted with 200 μL methanol and centrifugated again under the same conditions. The resultant supernatant was combined with the first one in the autosampler vial. Then, the autosampler vial was capped and the extracts were submitted for automated sample derivatization using multipurpose sampler MPS2 (Gerstel, Muehlheim, Germany). After sample preparation, microbial metabolites were quantitated with a gas chromatography coupled to time-of-flight mass spectrometry (GC-TOFMS) (Pegasus HT, Leco Corp, USA), followed by metabolite annotation and identification. The total mass of the metabolites was determined by metabolite diversity analysis. The reference library was developed consisting of 132 methyl and ethyl chloroformate (MCF and ECF) derivatized compounds with their mass spectral and retention index information for metabolite identification. The type of capillary column used for gas chromatography is Rxi-5MS which is 30 m (length) × 250 μm I.D and 0.25-μm film thickness.

### Data Analysis and Statistic Tests

Visualization and comparison of metabolite profile were performed by using principal component analysis model with permutation testing algorithm, to detect metabolic variation between the two groups. The univariate statistical analysis was also employed to perform the inter-group comparisons.

An R package named weighted gene co-expression network analysis (WGCNA) was applied to analyse the relationship between clinical traits of IBS and microbiological as well as clinical traits of IBS and metabolomic data. Initially, Pearson's correlation of each pair metabolite–microbe was calculated, and an adjacency matrix was constructed based on the Pearson's results and a predefined soft-thresholding parameter. A topology overlap matrix was calculated, and hierarchical clustering was performed to find the modules. Modules with a correlation coefficient >0.7 were merged into one module. The first eigenvector of the module was used to represent each module. To determine the membership of each gene in the module, the average connectivity of the gene in the module was calculated.

Spearman correlation was analyzed to evaluate the correlation between microbes, metabolites and pathophysiological features. *P*-values of <0.05 were considered as significant. A correlation network plot was generated, and correlation magnitudes > 0.6 (strong co-abundance relationships) and < −0.6 (strong co-exclusion relationships) were plotted. Visualization of the network was performed by Cytoscape v3.2.2.

## Results

### Patients Characteristics

Thirty adults were enrolled in this study, with 15 IBS patients meeting the Rome III criteria and another 15 healthy controls (HC). Among the IBS patients, 73.3% (11/15) had frequent abdominal pain (>10 times/week) and/or abdominal discomfort, and 60.0% (9/15) of them had increased bowel movements (3–5 times/day). Most of the IBS patients with abdominal pain may be relieved after bowel movement. All IBS patients enrolled in this study showed unformed (loose and watery) stools. The healthy controls had no abdominal pain and formed stool with normal bowel movements (Table 1).

**Table 1:** Demographic and clinical information of participants of this study.

	**IBS**	**Healthy people**
Number of patients	15	15
Mean age, year (SD)	47.67 (14.24)	28.27 (1.566)
Female, *n* (%)	5 (33.3%)	7 (46.7%)
Frequency of abdominal pain/week		
1–2 times *n* (%)	2 (13.3%)	0 (0.0%)
3–5 times *n* (%)	1 (6.7%)	0 (0.0%)
6–10 times *n* (%)	1 (6.7%)	0 (0.0%)
>10 times *n* (%)	11 (73.3%)	0 (0.0%)
Frequency of abdominal discomfort/week		
1–2 times *n* (%)	2 (13.3%)	2 (13.3%)
3–5 times *n* (%)	1 (6.7%)	0 (0.0%)
6–10 times *n* (%)	1 (6.7%)	0 (0.0%)
>10 times *n* (%)	11 (73.3%)	0 (0.0%)
Defecation times/day		
A (1–2 times) *n* (%)	6 (40.0%)	15 (100%)
B (3–5 times) *n* (%)	9 (60.0%)	0 (0.0%)
C (6–10 times) *n* (%)	0 (0.0%)	0 (0.0%)
D (>10 times) *n* (%)	0 (0.0%)	0 (0.0%)
Symptom duration		
1–2 years *n* (%)	9 (60.0%)	0 (0.0%)
3–5 years *n* (%)	3 (20%)	0 (0.0%)
>5 years *n* (%)	3 (20%)	0 (0.0%)
Stool property		
Unshaped stool *n* (%)	12 (93.3%)	0 (0.0%)
Mushy stool *n* (%)	3 (6.7%)	0 (0.0%)
Soft stool *n* (%)	0 (0.0%)	15 (100.0%)
Relief of abdominal pain after defecation		
Yes *n* (%)	14 (93.3%)	0 (0.0%)
No *n* (%)	1 (6.7%)	0 (0.0%)

### Metabolic Profiling Analysis in Different Groups

**Figure 1:**
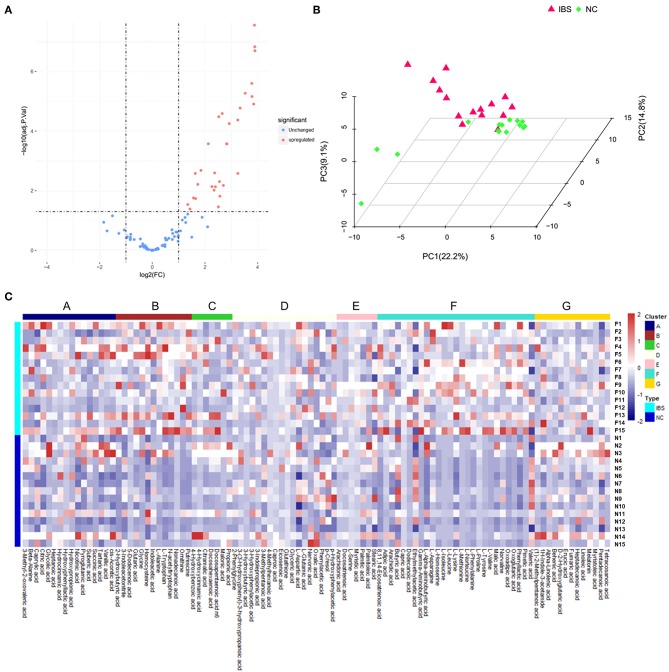
Metabolic profiling analysis in IBS and Healthy controls. **(A)** Volcano Plots revealed that there were 31 significantly up-regulated metabolites (red dots) in IBS patients compared to the healthy controls. **(B)** Principal component analysis (PCA) showed markedly different component profiles between the two groups. **(C)** The heatmap profile showed that all metabolites detected were divided into seven clusters. The clusters A–C, and F were significantly upregulated in the IBS group as compared to the healthy controls.

Metabolite analysis was conducted by using MicrobioMET 1.0 technology. The Volcano Plots revealed that there were 31 significantly up-regulated metabolites (red dots) in the IBS patient group when compared with the healthy controls (Figure 1A). Principal component analysis (PCA) showed markedly different component profiles between the two groups (Figure 1B). In addition, all metabolites detected were clustered, and a heat map profile was constructed (Figure 1C). The Figure S1A showed that all significantly differentially abundant metabolites were divided into seven clusters. The clusters A–C, and F were significantly up-regulated in the IBS group when compared with the healthy controls. The mean levels of these metabolites in different cluster, which were either amino acids or fatty acids were presented in Table 2 and Table S3. Taken together, these results indicated that the metabolites of IBS patients were significantly different from that of the healthy controls.

**Table 2:** Major differentially abundant fecal metabolites (abundance%) in IBS patients and healthy control.

**Metabolites**	**Irritable bowel syndrome (*n* = 15)^a^**	**Healthy controls (*n* = 15)^a^**	***P*-value^b^**	***Q*-value^c^**
8.11.14.Eicosatrienoic.acid	0.4015 (0.2588 to 0.7323)	0.02651 (0.01306 to 0.03774)	<0.0001	<0.0001
Capric.acid	0.01297 (0.005925 to 0.02282)	0.001884 (0.001097 to 0.003060)	<0.0001	<0.0001
Gamma.Aminobutyric.acid	0.9264 (0.5549 to 1.3016)	0.07469 (0.03423 to 0.1132)	<0.0001	0.0005
L. Homoserine	0.4402 (0.1948 to 0.7920)	0.06821 (0.04100 to 0.1421)	<0.0001	0.0005
L. Isoleucine	0.4013 (0.1336 to 1.1719)	0.02851 (0.01412 to 0.04918)	<0.0001	0.0005
L. Leucine	0.7815 (0.4295 to 2.2872)	0.04603 (0.02629 to 0.08831)	<0.0001	0.0005
L. Methionine	1.4862 (0.9226 to 11.4553)	0.1517 (0.1288 to 0.3844)	<0.0001	0.0005
L. Norleucine	0.2561 (0.1172 to 0.5144)	0.01578 (0.007898 to 0.02883)	<0.0001	0.0005
L. Phenylalanine	0.3267 (0.2405 to 0.7910)	0.03863 (0.01623 to 0.07230)	<0.0001	0.0005
L. Tryptophan	0.04805 (0.03404 to 0.09940)	0.01058 (0.006317 to 0.02460)	<0.0001	0.0005
L. Valine	0.5246 (0.2964 to 1.2419)	0.04274 (0.01852 to 0.06188)	<0.0001	0.0005
N. acetyltryptophan	0.06159 (0.05240 to 0.1753)	0.01155 (0.007426 to 0.02833)	<0.0001	0.0005
Oxoadipic.acid	1.7699 (0.8611 to 3.1822)	0.1248 (0.06129 to 0.3714)	<0.0001	0.0005
Putrescine	0.3064 (0.1681 to 0.5475)	0.01038 (0.004429 to 0.05220)	<0.0001	0.0005
Ornithine	0.2858 (0.1309 to 0.6154)	0.02619 (0.01670 to 0.03983)	0.0001	0.0005
Homocysteine	15.5655 (10.8261 to 29.2755)	3.4294 (0.7598 to 22.3389)	0.0186	0.0569

a *Concentrations are presented as median (interquartile range)*.

b *Obtained from Mann-Whitney U test*.

c *FDR adjusted p-value*.

### Microbial Profiling Analysis in Different Groups

**Figure 2:**
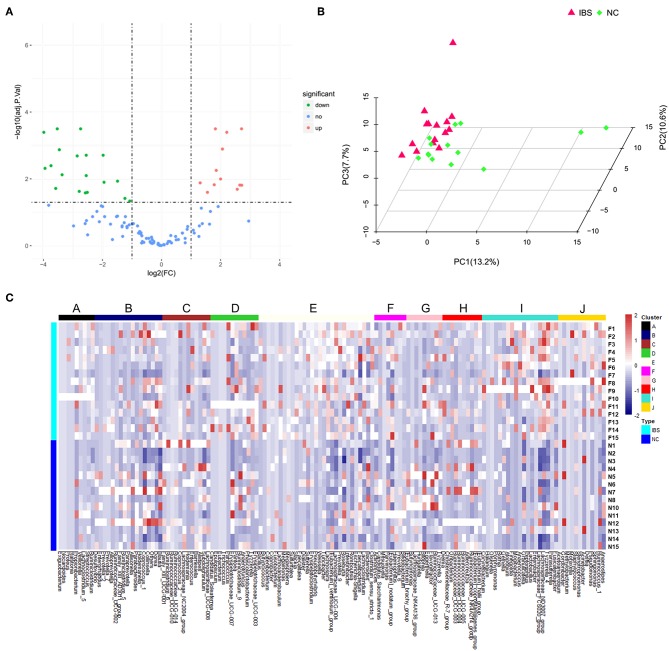
Microbial profiling analysis in IBS and healthy controls. **(A)** Volcano Plots showed that there were 19 significantly up-regulated (red dots) and 12 markedly down-regulated (green dots) microbial populations in IBS patients compared to the healthy controls. **(B)** PCA plots showed the IBS patients couldn't be discriminated from the healthy controls. **(C)** The heatmap profile showed that all microbes detected were divided into 10 clusters, and no single genus or genus cluster could clearly identify IBS from the healthy controls.

16S rDNA amplification and sequencing was performed to detect and identify fecal microbiota. Volcano Plots showed that there were 19 significantly up-regulated (red dots) and 12 markedly down-regulated (green dots) microbial populations between the IBS patients and the healthy controls (Figure 2A). The representative 15 significantly differential microbes between IBS patients and healthy controls were presented in Table 3. Analysis of the entry coordinates obtained from PCA plot indicated that fecal microbial profile was unable to differentiate IBS patients from healthy controls (Figure 2B). The heatmap profile showed all detected microbes (Figure 2C). The Figure S1B showed that all significantly differentially abundant microbes were divided into eight clusters. However, no single genus or cluster could clearly differentiate IBS patients from the healthy controls, indicating that the change of the gut microbiota in IBS patients was likely a comprehensive and personalized process.

**Table 3:** Major differentially abundant fecal microbes in IBS patients and healthy control.

**Taxonomy**	**Irritable bowel syndrome (*n* = 15)^a^**	**Healthy controls (*n* = 14)^a^**	***P*-value^b^**	***Q*-value^c^**
*Lachnoclostridium*	0.06487 (0.04371 to 0.1329)	0.02516 (0.01352 to 0.03376)	<0.0001	0.0015
*Clostridium_sensu_stricto_1*	0.03701 (0.01935 to 0.09547)	0.0008605 (0.0001639 to 0.003524)	<0.0001	0.0015
*Lachnospiraceae_UCG-004*	0.003274 (0.002821 to 0.004217)	0.0008195 (0.0002459 to 0.001065)	0.0001	0.0015
*Lactobacillus*	0.0007601 (0.0004166 to 0.001557)	0.000082 (0.0000 to 0.0002459)	0.0002	0.0021
*Tyzzerella_4*	0.003625 (0.002492 to 0.004231)	0.0003688 (0.0002459 to 0.0008195)	0.0002	0.0021
*Ruminiclostridium_5*	0.0003216 (0.0001535 to 0.0005408)	0.001106 (0.0007376 to 0.001721)	0.0003	0.0025
*Lachnospiraceae_ND3007_group*	0.002806 (0.001893 to 0.003661)	0.0003688 (0.00008200 to 0.001065)	0.0003	0.0025
*Ruminiclostridium*	0.003245 (0.002806 to 0.005467)	0.001803 (0.0009015 to 0.002295)	0.0004	0.0030
*Parabacteroides*	0.001374 (0.001169 to 0.002784)	0.01057 (0.003442 to 0.02049)	0.0006	0.0040
*Hungatella*	0.0000585 (0.00002920 to 0.00008770)	0.0004098 (0.0001639 to 0.0006556)	0.0019	0.0100
*Lachnospira*	0.004677 (0.002361 to 0.007272)	0.0009425 (0.0004098 to 0.003278)	0.0019	0.0100
*Lachnoclostridium_5*	0.002046 (0.00008772 to 0.003384)	0 (0.0000 to 0.0002459)	0.0050	0.0218
*Romboutsia*	0.03169 (0.01267 to 0.06433)	0.004303 (0.001393 to 0.02811)	0.0091	0.0336
*Lachnospiraceae_FCS020_group*	0.001988 (0.001352 to 0.002470)	0.0008605 (0.0003278 to 0.001311)	0.0100	0.0336
*Granulicatella*	0.0003508 (0.0001827 to 0.0004750)	0.000123 (0.0000 to 0.0003278)	0.0256	0.0663

a *Concentrations are presented as median (interquartile range)*.

b *Obtained from Mann-Whitney U test*.

c *FDR adjusted p-value*.

### The Association Between Metabolomic Profile and Severity of the IBS Symptom

**Figure 3:**
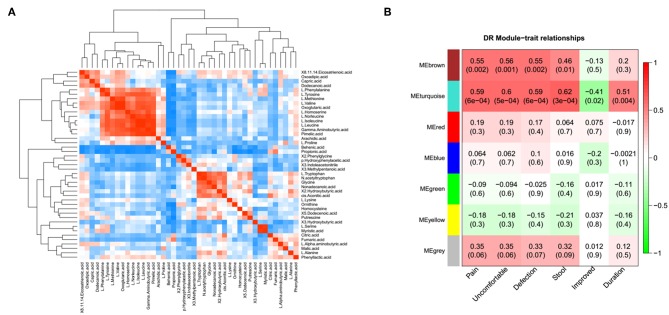
The association between metabolomic profile and severity of IBS. **(A)** The correlation matrix indicated that some metabolites were positively correlated with each other and some metabolites were negatively correlated with each other. Among them, partial amino acid is positively correlated with organic acid, such as L-Isoleucine with adipic acid. **(B)** Module-trait association of metabolites. Each row corresponds to a module, column to a trait. Each cell contains the corresponding correlation and *P*-value. Each table is colored by correlation according to the color legend on the left.

A correlation matrix was generated to test the association within differentially abundant metabolites. The correlation matrix revealed that some metabolites were positively correlated with each other while some others were negatively correlated with each other (Figure 3A). Among these, 10 amino acid were positively correlated with 5 organic acids, such as L-isoleucine with Gamma-Aminobutyric acid. In addition, the possible inherent association between differentially abundant metabolites and the severity of IBS symptoms was also investigated (Figure 3B). The correlation matrix showed that the module MEbrown of metabolites was positively associated with the frequency of the abdominal pain (*R* = 0.55, *p* = 0.002), the frequency of the abdominal discomfort (*R* = 0.56, *p* = 0.001), and defections (*R* = 0.55, *p* = 0.002). The module MEturquoise of metabolites was significantly associated with the frequency of abdominal pain (*R* = 0.59, *p* = 6e-04), the frequency of abdominal discomfort (*R* = 0.6, *p* = 5e-04), defections (*R* = 0.59, *p* = 6e-04), stool trait (*R* = 0.62, *p* = 3e-04), post-surgical relief of abdominal pain (*R* = −0.41, *p* = 0.02), and duration of symptoms (*R* = 0.51, *p* = 0.004) (Figure 3B). Representative metabolites in each module showed in Table S1 were significantly associated with the severity of IBS.

### The Association Between Microbial Profile and the Severity of IBS Symptom

**Figure 4:**
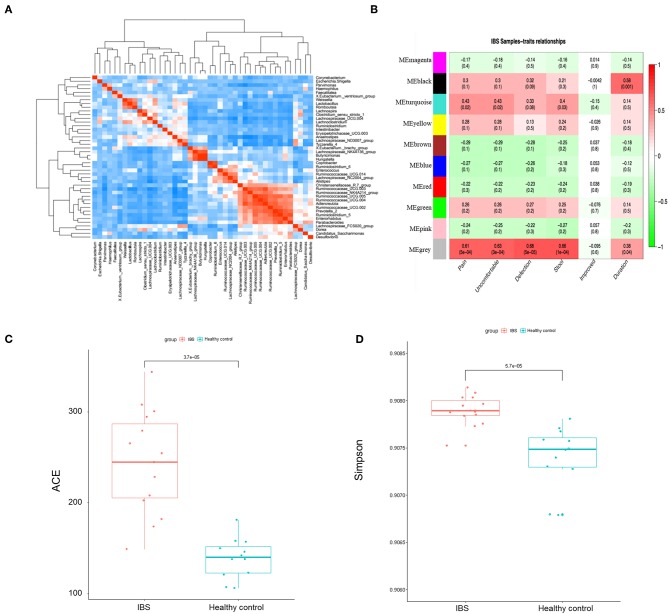
The association between microbial profile and severity of IBS. **(A)** The correlation matrix showed that some microbes were positively correlated with each other and some were negatively correlated with each other. **(B)** Module-trait association of microbes. Each row corresponds to a module of microbes, and column corresponds to a clinical trait. Each cell contains the corresponding correlation and *P*-value. Each table is colored by correlation according to the color legend on the left. Alpha diversity of ace **(C)** and simpson **(D)** index of the IBS patients and the healthy controls.

We then constructed the same correlation matrix to test the correlations within differentially abundant microbes (Figure 4A). The correlation matrix revealed that some microbes were positively correlated with each other and some microbes were negatively correlated with each other at genus level. For example, *Hungatella* was strongly correlated with *Butyricimonas* (*R* = 0.9, *P* < 0.001); *Anaerostipes* was negatively correlated with *Ruminococcaceae* (*R* = −0.44, *P* = 0.01). Furthermore, the possible inherent association between differentially abundant microbes and the severity of IBS was also investigated. As Figure 4B showed, the module MEblack of microbes was significantly associated with the duration of symptoms (*R* = 0.58, *p* = 0.001). The module MEturquoise of microbes was significantly associated with frequency of abdominal pain (*R* = 0.43, *p* = 0.02), frequency of abdominal discomfort (*R* = 0.43, *p* = 0.02), and stool trait (*R* = 0.4, *p* = 0.03). The module MEgrey was significantly associated with the frequency of abdominal pain (*R* = 0.61, *p* = 5e-04), the frequency of abdominal discomfort (*R* = 0.63, *p* = 3e-04), defections (*R* = 0.68, *p* = 5e-05), stool trait (*R* = 0.66, *p* = 1e-04), and the duration of symptoms (*R* = 0.38, *p* = 0.04) (Figure 4B). Representative microbes in each module showed in Table S2 were significantly associated with the severity of IBS. Microbial diversity (α-diversity and β-diversity) is reported to be involved in gut disorders. Therefore, the α-diversity index for fecal microbiota was calculated here. As shown in Figures 4C,D, both abundance-based coverage estimator (ACE) and simpson index indicated that the level of gut microbial diversity in the IBS group was significantly greater than that in healthy control.

### The Networks of Gut Microbiome and Metabolites in IBS Patients

**Figure 5:**
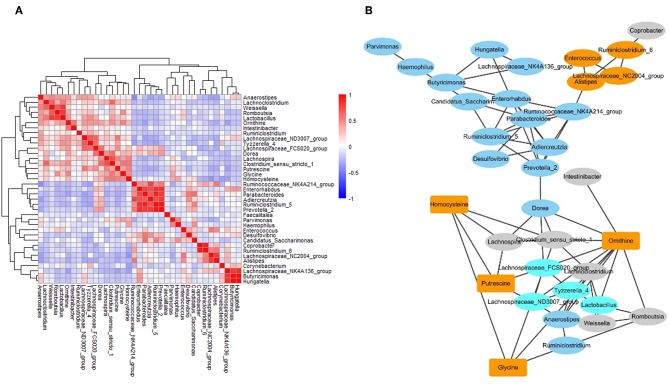
The networks of gut microbiome and metabolites in IBS patients. **(A)** The correlation matrices between microbial genus abundances and fecal metabolites. **(B)** A network generated by Cytoscape. Each node represented a metabolite/microbe. Ellipse node represent microbe, and rectangle node represent metabolite. Different color reflected different cluster by WGCNA. The edge linked the nodes reflected the Pearson correlation coefficient between those nodes. Higher coefficients were displayed with thicker lines.

To investigate the correlation between metabolites and microbes, a correlation matrix was generated by calculating the Pearson's correlation coefficient. Based on the correlation matrix (Figure 5A), some strong positive associations were discovered. For example, some amino acids were correlated with microbial genera, which may be due to their release through microbial proteolysis. In addition, a network generated in Cytoscape was used to visualize the correlation between metabolites and microbes (Figure 5B). The results revealed several significant metabolites–microbe relationships, including strongly positive association between glycine level and *Clostridium*, positive correlation of homocysteine with *Lachnospira, Clostridium* and *Haemophilus*, and negative association of homocysteine with *Corynebacterium* and *Lachnospiraceae*.

## Discussion

The underlying mechanisms contributed to IBS are thought to be heterogeneous. Many factors have been reported to play a role in the development of IBS, such as “brain-gut axis,” immune regulation, defection, risk gene mutations, and altered gastrointestinal motility (Louis et al., 2007; Holtmann et al., 2016; Defrees and Bailey, 2017). However, currently there are no clear IBS-related diagnostic biomarkers and the pathophysiology of IBS are still not fully understood. In this study, the fecal samples from IBS patients and healthy people were analyzed for the following three purposes: to get the quantitative metabolite and microbial signatures for IBS; to evaluate the possible microbe-metabolite associations; to investigate the relationship between the severity of IBS symptoms and metabolites as well as the severity of IBS symptoms and microbes. To our knowledge, this is the first study to analyze the networks of gut microbe-metabolite associations in fecal samples of adult IBS patients, and this is also the first report to determine the correlation of clinical characteristics with microbiota as well as clinical characteristics with metabolites in adult IBS patients.

In this study, we demonstrated that the fecal metabolome differed significantly between IBS patients and HC. The four clusters including 31 metabolites were significantly upregulated in the IBS group as compared to the healthy controls. Among these metabolites, we detected higher levels of L-methionine and homocysteine in IBS samples. Methionine and homocysteine are common amino acid obtained from food. Both methionine and homocysteine are generally considered to play an important role in intestinal health (Townsend et al., 2004). Homocysteine is an intermediary metabolite in methionine/cysteine metabolism. Consistent with our findings, Qian et al. reported that plasma homocysteine levels in IBS rats were significantly greater than in control rats and homocysteine-lowering treatment restored the barrier function in IBS mice (Zhao and Qian, 2014). These results suggested the role of homocysteine in disruption of the intestinal epithelial barrier in IBS patients.

Interestingly, we also found that some differentially abundant metabolites were positively correlated with each other and some were negatively correlated with each other, suggesting that further studies were needed to investigate the intrinsic links between these metabolites. For example, L-leucine was positively correlated with adipic acid (*R* = 0.83, *P* < 0.001). Adipic acid is a human xenobiotic metabolite and increased in urine of diabetes patients (Sonmez et al., 2007). Leucine is an essential amino acid in humans and contributes to regulation of blood-sugar levels. In addition, adipic acid and L-leucine have been identified as key factors of anti-inflammation mechanism of forsythiae fructus in rats (Yuan et al., 2017). Thus, those results suggested the positive associations between adipic acid and L-leucine might contribute to the inflammation process of IBS. Overall, the present study indicated that the changes of gut metabolites might play an important role in the development of IBS, and the gut metabolite might serve as the potential diagnostic biomarker of IBS.

In our study, 16S rDNA amplicon sequencing analysis showed that 19 microbial populations were significantly up-regulated, and 12 microbial populations were markedly down-regulated between IBS group and healthy controls. Among these gut microbes, the levels of *lachnospira* and *clostridium* were significantly increased in IBS patients than in healthy controls (3.8 and 30.0-fold, respectively). It is reported that several strains of *Lachnospira* were capable of producing lactate and acetate, which caused constipation by inhibiting mucin secretion (Canani et al., 2011). *Clostridium difficile*, one of the strain of *Clostridium*, was shown to increase the risk of post-infectious IBS, while the data available was scarce (Piche et al., 2007; Wadhwa et al., 2016). Unfortunately, no single cluster of microbes could be used to clearly identify IBS patients from healthy controls. This could be due to the various factors affecting the gut flora, which leads to the individual difference within each group. In addition, the results on association study of differentially abundant microbes showed the similar pattern as that of metabolites.

The intestinal metabolites or microbiota in IBS patients has also been investigated by other studies, and various alterations of specific metabolites or microbes have been reported (Krogius-Kurikka et al., 2009; Yu et al., 2018). However, the complex intestinal microbial communities showed in most of these previous studies were limited in resolution (Kerckhoffs et al., 2009). Even though several new studies have used omics analysis to investigate the characterization of the intestinal microbiota or metabolome (Rigsbee et al., 2012), these studies did not investigated the influence of metabolites or microbiota on the symptoms of IBS, as well as their correlation with each other (Carroll et al., 2012; Shankar et al., 2015).

Based on WGCNA, we obtained two modules of metabolites which were significantly associated with IBS clinical traits. Among these, the module turquoise was positively correlated with abdominal pain, duration of symptom, abdominal discomfort, and stool form. To our interest, we found that γ-aminobutyric acid (GABA) was included in module turquoise. It has been reported that the disruption of GABA leaded to neurological disease and enhancing GABA inhibition could alleviated sleep disorders, chronic pain and anxiety which have higher prevalence in IBS patients (Adeghate and Ponery, 2002; Raskov et al., 2016). Therefore, combined with our study, the results suggested the potential role of GABA in the treatment of visceral pain disorders. The results indicated a possible internal correlation between those clinical traits and metabolites.

The association studies of differentially abundant microbes and metabolites showed that microbes were significantly correlated with each other, and 4 metabolites (homocysteine, putrescine, glycine, and ornithine) were strongly associated with several microbes (*Clostridium, Lachnospira, Intestinibacter*, and et al.). These results suggested that the gut microbiota and metabolome were not only complex, but also interact with each other. In the present study, we found the presence of higher levels of several amino acids in IBS patients, which might suggest an active luminal proteolysis by gut microbiota in IBS patients. Indeed, some studies demonstrated that several strains of *Clostridium, Lachnospira*, and *Intestinibacter* could produce protease and belonged to proteolytic strains (Das Gupta and Sugiyama, 1972), which might explain our finding of positive correlation of amino acids with these microbes. These findings prompted that the changes of gut microbiota have impact on the change of gut metabolites, which provided a theoretical basis for future mechanism research on IBS.

Recently, we have reported a study on gut microbiota and metabolites of water-avoidance stress induced rats IBS model (Liu et al., 2019). Although the methods used in the present study were same as our previous study, the results of these two studies are not exactly consistent due to the difference in samples applied in the two researches. Consistently, both studies reported a significant difference in metabolite profile between the IBS and healthy controls, but no significantly differential microbial profiling. In addition, both studies showed the level of homocysteine elevated in the IBS group. Inconsistently, current study indicated that IBS patients exhibited an increased alpha diversity of the microbial population, which was different from the results of IBS rats in our previous study and some other studies. As for alterations of microbial diversity in patients with IBS, the results varies in different studies in which some presented a greater diversity (Zeber-Lubecka et al., 2016; Labus et al., 2017), some presented a lower diversity or no differences (Duan et al., 2019). Although α-diversity index is generally considered to be reduced in IBS patients, several studies reported an increased trend which indicated that factors influencing the nature of microbiome is complicated.

Inflammatory Bowel Disease (IBD) was also a common disorder of digestive tract. Studies have reported that IBD was also associated with the dramatic changes of gut microbiota and metabolome. Thus, both 16S rDNA amplification sequencing and metabolite profiling analyses were widely used in the research of IBD as well. Most studies on IBD have reported a decreased α-diversity of microbiota, which were similar with that of IBS (Dovrolis et al., 2019; Franzosa et al., 2019). In addition, short-chain fatty acids (SCFAs) were reported to be decreased in IBD patients, which is associated with intestinal microbial dysbiosis (Marchix et al., 2018). However, no significant difference on the level of SCFAs in IBS patients was reported. Although IBS was different from IBD, we could conclude that both gut microbiota and metabolome played an important role in the development of gastrointestinal disorders.

We acknowledged the following limitations in the present study. First, this is a single-center study with a limited sample size; however, it is comparable to previous studies that have attempted to illuminate the changes of metabolites or microbiota in IBS patients (Carroll et al., 2012; Liu et al., 2016). Second, significant differences in age between the two groups of participants may confound the results of the study as age might affect both microbiota structure and diversity. Thus, we analyzed the association of metabolites or microorganisms with age, and the results indicated that age was correlated with the module MEturquoise of metabolites (*R* = 0.4) or the module MEgrey of microbes (*R* = 0.44). Meanwhile, the module MEturquoise of metabolites was strongly correlated with the clinical traits including abdominal pain (*R* = 0.59), abdominal discomfort (*R* = 0.6), defection frequency (*R* = 0.59), and stool form (*R* = 0.62); the module MEgrey of microbes was strongly correlated with abdominal pain (*R* = 0.61), abdominal discomfort (*R* = 0.63), defection frequency (*R* = 0.68), and stool form (*R* = 0.66). The results indicated that although age was associated with some of the metabolites, the correlation was weaker than the correlation between clinical symptoms and metabolites as well as clinical symptoms and microbes (R is <0.6). Therefore, the observed differences in the metabolome or microbiome between IBS patients and healthy controls mainly attributed to the development of IBS rather than the effect of age. Afterall, all above factors need to be considered in future studies.

Taken together, in this exploratory study, we identified specific metabolites or microbes in fecal samples that could be potential biomarkers for differentiation IBS patients from healthy controls. We also found that some fecal metabolites or microbes were associated with the clinical traits of IBS. In addition, some of these metabolites were strongly associated with microbes, suggesting these metabolites were likely to be originated from microbial metabolism. However, further studies are needed to investigate the members of differential abundant metabolites and microbiota; It is also important to elucidate their roles in gut metabolic processes and their potential interplay with inflammation and nervous systems.

## Data Availability Statement

The datasets generated for this study can be found in Sequence Read Archive using the accession number PRJNA566284.

## Ethics Statement

The studies involving human participants were reviewed and approved by the Ethics Committee of Beijing Friendship Hospital. The patients/participants provided their written informed consent to participate in this study.

## Author Contributions

SZha and LM: designed the experiments. SZhu, SL, LC, HL, and ZZ: performed the experiments. SL, LM, JG, YZ, and QZ: analyzed the data. SL and SZhu: wrote the manuscript. SZha and LM: revised the manuscript. All authors have approved the final version of the manuscript and agree to be accountable for all aspects of the work, ensuring that questions related to the accuracy or integrity of any part of the work are appropriately investigated and resolved.

### Conflict of Interest

The authors declare that the research was conducted in the absence of any commercial or financial relationships that could be construed as a potential conflict of interest.

## Supplementary Material

Figure S1 The heatmap of significantly differential abundant metabolites or microbes. **(A)** The heatmap profile with the statistically significant metabolites (*P* < 0.05). **(B)** The heatmap profile with the statistically significant microbes (*P* < 0.05).

Table S1 Correlation of fecal metabolites with clinical traits of IBS.

Table S2 Correlation of fecal microbes with clinical traits of IBS.

Table S3 Major differentially abundant fecal metabolites in IBS patients and healthy control.

## References

[B1] Adeghate E. Ponery A. S. (2002). GABA in the endocrine pancreas: cellular localization and function in normal and diabetic rats. Tissue Cell 34, 1–6. 10.1054/tice.2002.0217 11989965

[B2] Belizario J. E. Faintuch J. (2018). Microbiome and gut dysbiosis. Exp. Suppl. 109, 459–476. 10.1007/978-3-319-74932-7_13 30535609

[B3] Canani R. B. Costanzo M. D. Leone L. Pedata M. Meli R. Calignano A. (2011). Potential beneficial effects of butyrate in intestinal and extraintestinal diseases. World J. Gastroenterol. 17, 1519–1528. 10.3748/wjg.v17.i12.1519 21472114 PMC3070119

[B4] Carroll I. M. Ringel-Kulka T. Siddle J. P. Ringel Y. (2012). Alterations in composition and diversity of the intestinal microbiota in patients with diarrhea-predominant irritable bowel syndrome. Neurogastroenterol. Motil. 24, 521–530. e248. 10.1111/j.1365-2982.2012.01891.x 22339879 PMC3975596

[B5] Costello E. K. Lauber C. L. Hamady M. Fierer N. Gordon J. I. Knight R. (2009). Bacterial community variation in human body habitats across space and time. Science 326, 1694–1697. 10.1126/science.1177486 19892944 PMC3602444

[B6] Das Gupta B. R. Sugiyama H. (1972). Role of a protease in natural activation of *Clostridium botulinum* neurotoxin. Infect. Immun. 6, 587–590. 4564288 10.1128/iai.6.4.587-590.1972 PMC422579

[B7] Defrees D. N. Bailey J. (2017). Irritable bowel syndrome: epidemiology, pathophysiology, diagnosis, and treatment. Prim. Care 44, 655–671. 10.1016/j.pop.2017.07.009 29132527

[B8] Dovrolis N. Drygiannakis I. Filidou E. Kandilogiannakis L. Arvanitidis K. Tentes I.. (2019). Gut microbial signatures underline complicated crohn's disease but vary between cohorts; an *in silico* approach. Inflamm. Bowel Dis. 25, 217–225. 10.1093/ibd/izy328 30346536

[B9] Drossman D. A. McKee D. C. Sandler R. S. Mitchell C. M. Cramer E. M. Lowman B. C.. (1988). Psychosocial factors in the irritable bowel syndrome. A multivariate study of patients and nonpatients with irritable bowel syndrome. Gastroenterology 95, 701–708. 10.1016/S0016-5085(88)80017-9 3396817

[B10] Drossman D. A. Patrick D. L. Whitehead W. E. Toner B. B. Diamant N. E. Hu Y.. (2000). Further validation of the IBS-QOL: a disease-specific quality-of-life questionnaire. Am. J. Gastroenterol. 95, 999–1007. 10.1111/j.1572-0241.2000.01941.x 10763950

[B11] Duan R. Zhu S. Wang B. Duan L. (2019). Alterations of gut microbiota in patients with irritable bowel syndrome based on 16S rRNA-targeted sequencing: a systematic review. Clin. Transl. Gastroenterol. 10:e00012. 10.14309/ctg.0000000000000012 30829919 PMC6407812

[B12] Ford A. C. Lacy B. E. Talley N. J. (2017). Irritable bowel syndrome. N. Engl. J. Med. 376, 2566–2578. 10.1056/NEJMra1607547 28657875

[B13] Franzosa E. A. Sirota-Madi A. Avila-Pacheco J. Fornelos N. Haiser H. J. Reinker S. (2019). Gut microbiome structure and metabolic activity in inflammatory bowel disease. Nat. Microbiol. 4, 293–305. 10.1038/s41564-018-0306-4 30531976 PMC6342642

[B14] Ghaisas S. Maher J. Kanthasamy A. (2016). Gut microbiome in health and disease: linking the microbiome-gut-brain axis and environmental factors in the pathogenesis of systemic and neurodegenerative diseases. Pharmacol. Ther. 158, 52–62. 10.1016/j.pharmthera.2015.11.012 26627987 PMC4747781

[B15] Guilera M. Balboa A. Mearin F. (2005). Bowel habit subtypes and temporal patterns in irritable bowel syndrome: systematic review. Am. J. Gastroenterol. 100, 1174–1184. 10.1111/j.1572-0241.2005.40674.x 15842596

[B16] Holtmann G. J. Ford A. C. Talley N. J. (2016). Pathophysiology of irritable bowel syndrome. Lancet Gastroenterol. Hepatol. 1, 133–146. 10.1016/S2468-1253(16)30023-1 28404070

[B17] Kellow J. E. Phillips S. F. (1987). Altered small bowel motility in irritable bowel syndrome is correlated with symptoms. Gastroenterology 92, 1885–1893. 10.1016/0016-5085(87)90620-2 3569764

[B18] Kerckhoffs A. P. Samsom M. van der Rest M. E. de Vogel J. Knol J. Ben-Amor K.. (2009). Lower Bifidobacteria counts in both duodenal mucosa-associated and fecal microbiota in irritable bowel syndrome patients. World J. Gastroenterol. 15, 2887–2892. 10.3748/wjg.15.2887 19533811 PMC2699007

[B19] Krogius-Kurikka L. Lyra A. Malinen E. Aarnikunnas J. Tuimala J. Paulin L.. (2009). Microbial community analysis reveals high level phylogenetic alterations in the overall gastrointestinal microbiota of diarrhoea-predominant irritable bowel syndrome sufferers. BMC Gastroenterol. 9:95. 10.1186/1471-230X-9-95 20015409 PMC2807867

[B20] Labus J. S. Hollister E. B. Jacobs J. Kirbach K. Oezguen N. Gupta A.. (2017). Differences in gut microbial composition correlate with regional brain volumes in irritable bowel syndrome. Microbiome 5:49. 10.1186/s40168-017-0260-z 28457228 PMC5410709

[B21] Liu S. Si C. Yu Y. Zhao G. Chen L. Zhao Y.. (2019). Multi-omics analysis of gut microbiota and metabolites in rats with irritable bowel syndrome. Front. Cell. Infect. Microbiol. 9:178. 10.3389/fcimb.2019.00178 31192167 PMC6549239

[B22] Liu Y. Zhang L. Wang X. Wang Z. Zhang J. Jiang R.. (2016). Similar fecal microbiota signatures in patients with diarrhea-predominant irritable bowel syndrome and patients with depression. Clin. Gastroenterol. Hepatol. 14, 1602–1611.e1605. 10.1016/j.cgh.2016.05.033 27266978

[B23] Lopetuso L. R. Petito V. Graziani C. Schiavoni E. Paroni Sterbini F. Poscia A.. (2018). Gut microbiota in health, diverticular disease, irritable bowel syndrome, and inflammatory bowel diseases: time for microbial marker of gastrointestinal disorders. Dig. Dis. 36, 56–65. 10.1159/000477205 28683448

[B24] Louis P. Scott K. P. Duncan S. H. Flint H. J. (2007). Understanding the effects of diet on bacterial metabolism in the large intestine. J. Appl. Microbiol. 102, 1197–1208. 10.1111/j.1365-2672.2007.03322.x 17448155

[B25] Lovell R. M. Ford A. C. (2012a). Effect of gender on prevalence of irritable bowel syndrome in the community: systematic review and meta-analysis. Am. J. Gastroenterol. 107, 991–1000. 10.1038/ajg.2012.131 22613905

[B26] Lovell R. M. Ford A. C. (2012b). Global prevalence of and risk factors for irritable bowel syndrome: a meta-analysis. Clin. Gastroenterol. Hepatol. 10, 712–721.e714. 10.1016/j.cgh.2012.02.029 22426087

[B27] Marchix J. Goddard G. Helmrath M. A. (2018). Host-gut microbiota crosstalk in intestinal adaptation. Cell Mol. Gastroenterol. Hepatol. 6, 149–162. 10.1016/j.jcmgh.2018.01.024 30023411 PMC6047313

[B28] Mayer E. A. (2008). Clinical practice. Irritable bowel syndrome. N. Engl. J. Med. 358, 1692–1699. 10.1056/NEJMcp0801447 18420501 PMC3816529

[B29] Mayer E. A. Tillisch K. (2011). The brain-gut axis in abdominal pain syndromes. Annu. Rev. Med. 62, 381–396. 10.1146/annurev-med-012309-103958 21090962 PMC3817711

[B30] Noorbakhsh H. Yavarmanesh M. Mortazavi S. A. Adibi P. Moazzami A. A. (2018). Metabolomics analysis revealed metabolic changes in patients with diarrhea-predominant irritable bowel syndrome and metabolic responses to a synbiotic yogurt intervention. Eur. J. Nutr. 10.1007/s00394-018-1855-2. [Epub ahead of print]. 30392136

[B31] Ohman L. Simren M. (2010). Pathogenesis of IBS: role of inflammation, immunity and neuroimmune interactions. Nat. Rev. Gastroenterol. Hepatol. 7, 163–173. 10.1038/nrgastro.2010.4 20101257

[B32] O'Malley D. (2019). Endocrine regulation of gut function - a role for glucagon-like peptide-1 in the pathophysiology of irritable bowel syndrome. Exp. Physiol. 104, 3–10. 10.1113/EP087443 30444291

[B33] Oswiecimska J. Szymlak A. Roczniak W. Girczys-Poledniok K. Kwiecien J. (2017). New insights into the pathogenesis and treatment of irritable bowel syndrome. Adv. Med. Sci. 62, 17–30. 10.1016/j.advms.2016.11.001 28135659

[B34] Piche T. Vanbiervliet G. Pipau F. G. Dainese R. Hebuterne X. Rampal P.. (2007). Low risk of irritable bowel syndrome after *Clostridium difficile* infection. Can. J. Gastroenterol. 21, 727–731. 10.1155/2007/262478 18026576 PMC2658587

[B35] Raskov H. Burcharth J. Pommergaard H. C. Rosenberg J. (2016). Irritable bowel syndrome, the microbiota and the gut-brain axis. Gut Microbes 7, 365–383. 10.1080/19490976.2016.1218585 27472486 PMC5046167

[B36] Rigsbee L. Agans R. Shankar V. Kenche H. Khamis H. J. Michail S.. (2012). Quantitative profiling of gut microbiota of children with diarrhea-predominant irritable bowel syndrome. Am. J. Gastroenterol. 107, 1740–1751. 10.1038/ajg.2012.287 22986438

[B37] Rogers J. Henry M. M. Misiewicz J. J. (1989). Increased segmental activity and intraluminal pressures in the sigmoid colon of patients with the irritable bowel syndrome. Gut 30, 634–641. 10.1136/gut.30.5.634 2731756 PMC1434217

[B38] Shankar V. Homer D. Rigsbee L. Khamis H. J. Michail S. Raymer M.. (2015). The networks of human gut microbe-metabolite associations are different between health and irritable bowel syndrome. ISME J. 9, 1899–1903. 10.1038/ismej.2014.258 25635640 PMC4511929

[B39] Si J. M. Yu Y. C. Fan Y. J. Chen S. J. (2004). Intestinal microecology and quality of life in irritable bowel syndrome patients. World J. Gastroenterol. 10, 1802–1805. 10.3748/wjg.v10.i12.1802 15188510 PMC4572273

[B40] Sonmez G. Mutlu H. Ozturk E. Sildiroglu H. O. Keskin A. T. Basekim C. C.. (2007). Magnetic resonance imaging findings of adult-onset glutaric aciduria type I. Acta Radiol. 48, 557–559. 10.1080/02841850701280874 17520433

[B41] Talley N. J. Boyce P. M. Jones M. (1997). Predictors of health care seeking for irritable bowel syndrome: a population based study. Gut 41, 394–398. 10.1136/gut.41.3.394 9378398 PMC1891476

[B42] Townsend D. M. Tew K. D. Tapiero H. (2004). Sulfur containing amino acids and human disease. Biomed. Pharmacother. 58, 47–55. 10.1016/j.biopha.2003.11.005 14739061 PMC6361141

[B43] Wadhwa A. Al Nahhas M. F. Dierkhising R. A. Patel R. Kashyap P. Pardi D. S.. (2016). High risk of post-infectious irritable bowel syndrome in patients with *Clostridium difficile* infection. Aliment. Pharmacol. Ther. 44, 576–582. 10.1111/apt.13737 27444134 PMC4982831

[B44] Yamashiro Y. (2017). Gut microbiota in health and disease. Ann. Nutr. Metab. 71, 242–246. 10.1159/000481627 29136611

[B45] Yu L. M. Zhao K. J. Wang S. S. Wang X. Lu B. (2018). Gas chromatography/mass spectrometry based metabolomic study in a murine model of irritable bowel syndrome. World J. Gastroenterol. 24, 894–904. 10.3748/wjg.v24.i8.894 29491683 PMC5829153

[B46] Yuan A. Gong L. Luo L. Dang J. Gong X. Zhao M.. (2017). Revealing anti-inflammation mechanism of water-extract and oil of forsythiae fructus on carrageenan-Induced edema rats by serum metabolomics. Biomed. Pharmacother. 95, 929–937. 10.1016/j.biopha.2017.09.009 28915534

[B47] Zeber-Lubecka N. Kulecka M. Ambrozkiewicz F. Paziewska A. Goryca K. Karczmarski J.. (2016). Limited prolonged effects of rifaximin treatment on irritable bowel syndrome-related differences in the fecal microbiome and metabolome. Gut Microbes 7, 397–413. 10.1080/19490976.2016.1215805 27662586 PMC5046165

[B48] Zhao Y. Qian L. (2014). Homocysteine-mediated intestinal epithelial barrier dysfunction in the rat model of irritable bowel syndrome caused by maternal separation. Acta Biochim. Biophys. Sin. 46, 917–919. 10.1093/abbs/gmu076 25187412

