# Supplementary figures and images for: Identification of Gut Microbiota and Metabolites Signature in Patients With Irritable Bowel Syndrome
Source: Front Cell Infect Microbiol. 2019 Oct 18;9:346. doi: 10.3389/fcimb.2019.00346 (PMC6813219; doi:10.3389/fcimb.2019.00346)

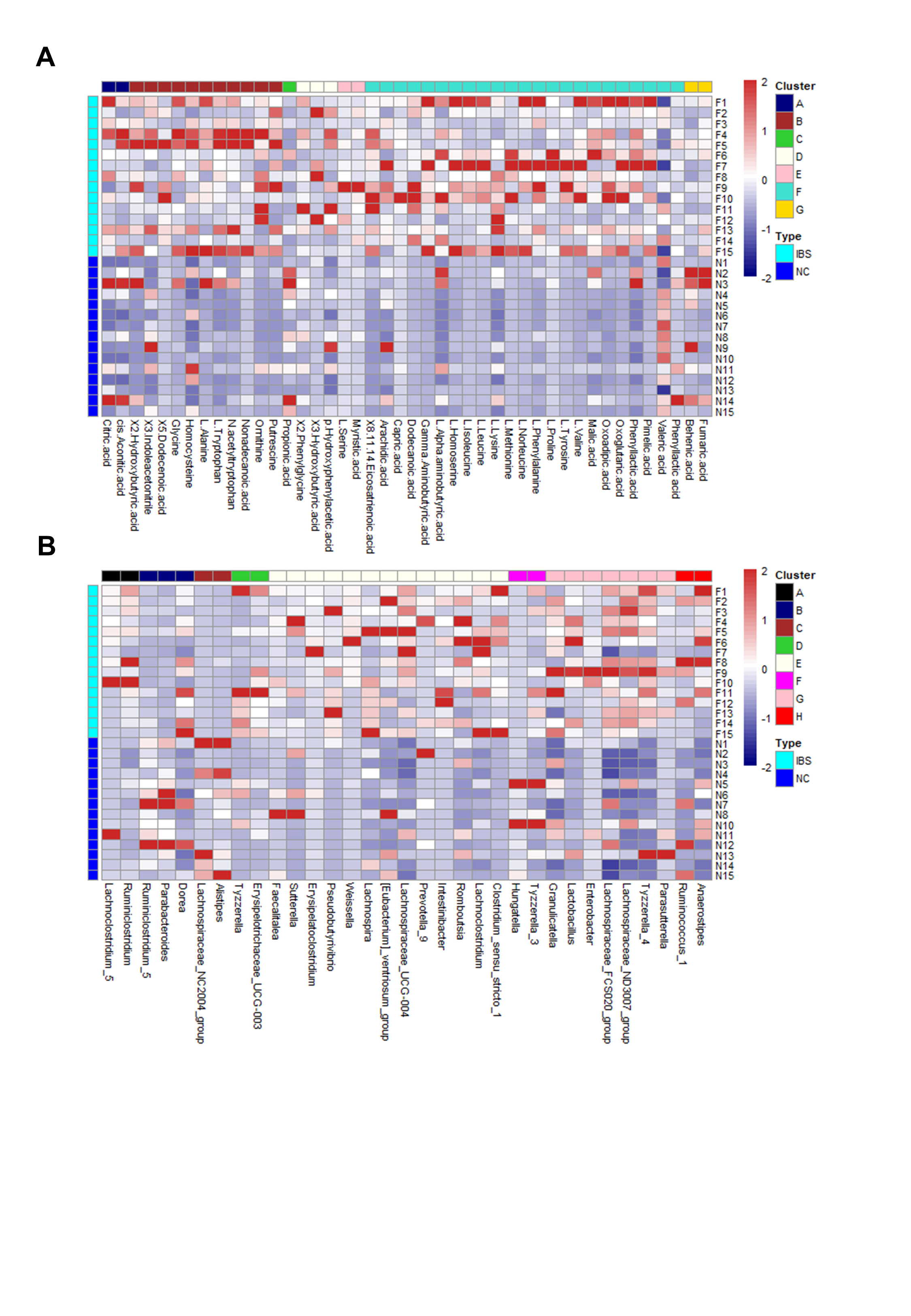

Supplement: Figure S1 — The heatmap of significantly differential abundant metabolites or microbes. (A) The heatmap profile with the statistically significant metabolites (P < 0.05). (B) The heatmap profile with the statistically significant microbes (P < 0.05). [file Image_1.TIF]
