# Supplementary material for: Identification of Gut Microbiota and Metabolites Signature in Patients With Irritable Bowel Syndrome
Source: Front Cell Infect Microbiol. 2019 Oct 18;9:346. doi: 10.3389/fcimb.2019.00346 (PMC6813219; doi:10.3389/fcimb.2019.00346)
Supplement: Table S1 — Correlation of fecal metabolites with clinical traits of IBS. [file Data_Sheet_1.doc]

**Table S1 Correlation of fecal metabolites with clinical traits of IBS**

| Module | Metabolites | Abdominal pain | Duration of symptoms | Abdominal discomfort | Stool trait |
| --- | --- | --- | --- | --- | --- |
| Brown | Ornithine  Putrescine  N-acetyltryptophan  L-Tryptophan | R=0.55，P=0.02 | R=0.2, p=0.3 | R=0.56, p=0.002 | R=0.46, p=0.01 |
| Turquoise | 8,11,14-Eicosatrienoic acid  Oxoadipic acid  L-Phenylalanine  L-Valine  Gamma-Aminobutyric acid | R=0.59，P=6e-04 | R=0.51, p=0.04 | R=0.6, p=5e-04 | R=0.62, P=3e-04 |
