# Supplementary material for: Identification of Gut Microbiota and Metabolites Signature in Patients With Irritable Bowel Syndrome
Source: Front Cell Infect Microbiol. 2019 Oct 18;9:346. doi: 10.3389/fcimb.2019.00346 (PMC6813219; doi:10.3389/fcimb.2019.00346)
Supplement: Table S2 — Correlation of fecal microbes with clinical traits of IBS. [file Data_Sheet_2.doc]

**Table S2**

**Correlation of fecal microbes with clinical traits of IBS**

| Module | Metabolites | Abdominal pain | Duration of symptoms | Abdominal discomfort | Stool trait |
| --- | --- | --- | --- | --- | --- |
| Grey | *Lachnoclostridium*  *Romboutsia*  *Clostridium_sensu_stricto_1*  *Lachnospira*  *Lachnospiraceae_UCG-004* | R=0.61, P=5e-04 | P=0.38, P=0.04 | R=0.63, P=3e-04 | R=0.66, P=1e-04 |
| Turquoise | *Tyzzerella_4*  *Lachnospiraceae_FCS020_group*  *Lachnospiraceae_ND3007_group*  *Lactobacillus*  *Granulicatella* | R=0.43, P=0.02 | R=0.14, P=0.5 | R=0.43, P=0.02 | R=0.4, P=0.03 |
| Black | *Lachnoclostridium_5*  *Ruminiclostridium*  *Veillonella*  *Streptococcus* | R=0.3, P=0.1 | R=0.58, P=0.001 | R=0.3, P=0.1 | R=0.21 P=0.3 |
