# Supplementary material for: Identification of Gut Microbiota and Metabolites Signature in Patients With Irritable Bowel Syndrome
Source: Front Cell Infect Microbiol. 2019 Oct 18;9:346. doi: 10.3389/fcimb.2019.00346 (PMC6813219; doi:10.3389/fcimb.2019.00346)
Supplement: Table S3 — Major differentially abundant fecal metabolites in IBS patients and healthy control. [file Data_Sheet_3.doc]

**Table S3** Major differentially abundant fecal metabolites in IBS patients and healthy control

| **Metabolites** | **Irritable bowel syndrome** **(n=15) ^a^** | **Healthy controls** **(n=15) ^a^** | **P value^b^** | **Q value^c^** |
| --- | --- | --- | --- | --- |
| 8.11.14.Eicosatrienoic.acid | 0.4015（0.2588 to 0.7323） | 0.02651（0.01306 to 0.03774） | < 0.0001 | < 0.0001 |
| Capric.acid | 0.01297（0.005925 to 0.02282） | 0.001884（0.001097 to 0.003060） | < 0.0001 | < 0.0001 |
| Gamma.Aminobutyric.acid | 0.9264（0.5549 to 1.3016） | 0.07469（0.03423 to 0.1132） | < 0.0001 | 0.0005 |
| L.Homoserine | 0.4402（0.1948 to 0.7920） | 0.06821（0.04100 to 0.1421） | < 0.0001 | 0.0005 |
| L.Isoleucine | 0.4013（0.1336 to 1.1719） | 0.02851（0.01412 to 0.04918） | < 0.0001 | 0.0005 |
| L.Leucine | 0.7815（0.4295 to 2.2872） | 0.04603（0.02629 to 0.08831） | < 0.0001 | 0.0005 |
| L.Methionine | 1.4862（0.9226 to 11.4553） | 0.1517（0.1288 to 0.3844） | < 0.0001 | 0.0005 |
| L.Norleucine | 0.2561（0.1172 to 0.5144） | 0.01578（0.007898 to 0.02883） | < 0.0001 | 0.0005 |
| L.Phenylalanine | 0.3267（0.2405 to 0.7910） | 0.03863（0.01623 to 0.07230） | < 0.0001 | 0.0005 |
| L.Tryptophan | 0.04805（0.03404 to 0.09940） | 0.01058（0.006317 to 0.02460） | < 0.0001 | 0.0005 |
| L.Tyrosine | 0.4863（0.1407 to 0.9898） | 0.03148（0.02148 to 0.07783） | < 0.0001 | 0.0005 |
| L.Valine | 0.5246（0.2964 to 1.2419） | 0.04274（0.01852 to 0.06188） | < 0.0001 | 0.0005 |
| N.acetyltryptophan | 0.06159（0.05240 to 0.1753） | 0.01155（0.007426 to 0.02833） | < 0.0001 | 0.0005 |
| Oxoadipic.acid | 1.7699（0.8611 to 3.1822） | 0.1248（0.06129 to 0.3714） | < 0.0001 | 0.0005 |
| Oxoglutaric.acid | 0.8755（0.4654 to 2.5041） | 0.08072（0.03498 to 0.1141） | < 0.0001 | 0.0005 |
| Pimelic.acid | 0.17（0.07041 to 0.3639） | 0.01623（0.002723 to 0.01861） | < 0.0001 | 0.0005 |
| Putrescine | 0.3064（0.1681 to 0.5475） | 0.01038（0.004429 to 0.05220） | < 0.0001 | 0.0005 |
| Ornithine | 0.2858（0.1309 to 0.6154） | 0.02619（0.01670 to 0.03983） | 0.0001 | 0.0005 |
| Homocysteine | 15.5655（10.8261 to 29.2755）  ( | 3.4294（0.7598 to 22.3389） | 0.0186 | 0.0569 |
| Myristic.acid | 0.2227（0.1597 to 0.4106） | 0.07295（0.04413 to 0.1012） | 0.0001 | 0.0005 |
| X2.Hydroxybutyric.acid | 0.006792（0.003920 to 0.01085） | 0.0004519（0.0002507 to 0.001055） | 0.0002 | 0.0010 |
| Phenyllactic.acid | 0.00656（0.004325 to 0.01079） | 0.0006064（0.0003108 to 0.002509） | 0.0003 | 0.0014 |
| L.Lysine | 1.1869（0.7401 to 1.9361） | 0.2765（0.2287 to 0.9024） | 0.0006 | 0.0026 |
| Dodecanoic.acid | 0.2034（0.06971 to 0.6778） | 0.01326（0.006571 to 0.02631） | 0.0008 | 0.0034 |
| L.Alanine | 0.01958（0.008127 to 0.03983）  0.008127 to 0.03983  ） | 0.003621（0.001136 to 0.005706） | 0.0012 | 0.0047 |
| Malic.acid | 0.0627（0.03575 to 0.09791） | 0.01964（0.009145 to 0.03573） | 0.0012 | 0.0047 |
| L.Proline | 0.1034（0.08198 to 0.2146） | 0.05874（0.03639 to 0.07341） | 0.0017 | 0.0064 |
| Glycine | 0.07058（0.01392 to 0.2097） | 0.007194（0.003505 to 0.01180） | 0.0037 | 0.0133 |
| Nonadecanoic.acid | 0.007465（0.002672 to 0.01834） | 0.001334（0.0006045 to 0.002389） | 0.0043 | 0.0150 |
| L.Alpha.aminobutyric.acid | 0.02214（0.01582 to 0.04203） | 0.002673（0.001274 to 0.02016） | 0.0075 | 0.0253 |
| L.Serine | 0.1025（0.02758 to 0.2364） | 0.02586（0.01049 to 0.03311） | 0.0099 | 0.0323 |

^a^ Concentrations are presented as median (interquartile range).

^b^ Obtained from Mann‐Whitney U test

^c^ FDR adjusted p-value
